# New Frameshifting Pseudoknot Found in SARS Virus

**DOI:** 10.1371/journal.pbio.0030199

**Published:** 2005-05-17

Viruses, small organisms that hijack our cellular machinery to replicate their genomes and make new viruses, constantly threaten human health. Not only are we unable to control infections caused by old enemies such as the cold virus, but we are also continually challenged by new enemies, like the coronavirus that causes severe acute respiratory syndrome (SARS-CoV). Vaccines provide one way to deal with viruses, but subtle differences between how host and viral proteins are made may also provide targets for new antiviral therapeutics.

SARS, a life-threatening respiratory illness, first appeared in late 2002. By February 2003, Guangdong Province, China, was in the grip of a SARS epidemic, and public-health officials were predicting that millions of people might become infected. The rapid implementation of effective containment efforts averted this new threat to human health, and, in the end, only 8,098 people became ill. SARS-CoV, a single-stranded RNA virus, was isolated in March 2003 and its genome sequenced by May 2003. Since then, researchers have intensively studied the virus, hoping to identify targets for antiviral therapeutics. Jonathan Dinman and colleagues now describe a new RNA structural motif in the SARS-CoV genome that may provide such a target.

During protein synthesis, molecular machines called ribosomes move along mRNA molecules, translating nucleotide triplets into amino acids. In human cells, the ribosomes usually hook onto the start of an mRNA and decode each triplet in turn. However, viral mRNAs often contain special signals that tell the ribosomes to change register or “frameshift.” This allows viruses to coordinate gene expression from overlapping coding sequences, and it ensures that the correct ratios of enzymatic and structural proteins are made.[Fig pbio-0030199-g001]

**Figure pbio-0030199-g001:**
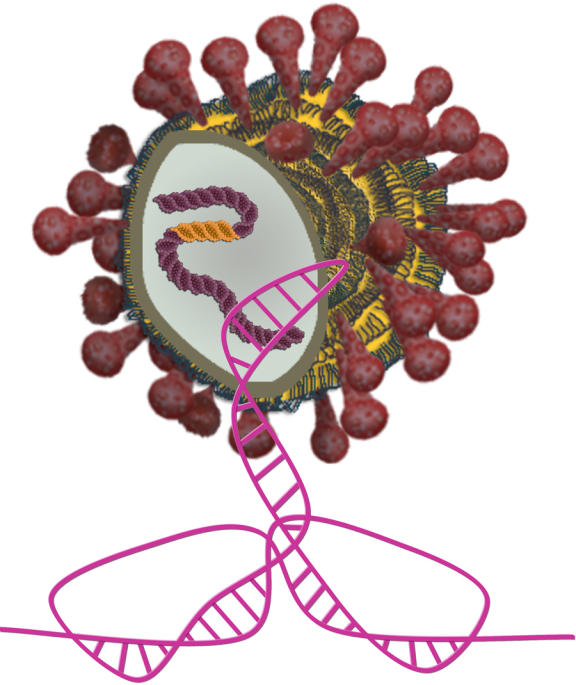
A three-stemmed pseudoknot in SARS messenger RNAs could provide a target for antiviral therapeutics (Image: Jonathan Dinman and the National Institute of Allergy and Infectious Diseases)

ORF1a and ORF1b are overlapping, out-of-frame coding sequences within the SARS-CoV genome. Each encodes a polyprotein—a large protein that is cleaved into smaller, functional proteins. Polyprotein 1a is translated directly from ORF1a; the fused polyprotein 1a/1b is produced by programmed −1 ribosomal frameshifting in which the ribosome slips back one nucleotide at a special signal within the mRNA. Like other frameshift signals, the SARS-CoV signal contains a pseudoknot, a stable mRNA structure. Pseudoknots generally contain two stems, in which complementary nucleotides form double-stranded RNA, and two or three loops of unpaired nucleotides. Because the mRNA strand passes over and behind itself to form the stems, the whole structure looks like a small knot of the kind that would unravel if its two ends were pulled.

Unexpectedly, a computational analysis undertaken by Dinman and his colleagues reveals that the pseudoknot in the SARS-CoV frameshift signal contains three stems. The researchers provide further evidence for this novel structure by finding potential three-stemmed pseudoknots in the frameshift signals of other coronaviruses and by doing biochemical and structural NMR studies on the SARS-CoV signal. They also show that the SARS-CoV frameshift signal behaves like other viral frameshift signals in several frameshifting assays, and their mutagenesis studies indicate that specific sequences and structures within stem 2 of the pseudoknot are needed for efficient frameshifting.

The exact role of the extra stem in the SARS-CoV frameshifting signal remains to be determined, but the researchers speculate that it could help to regulate the exact ratio of polyprotein 1a to 1a/1b. The current results also suggest that the three stems may fold back on one another to from a complex globular RNA structure. The elucidation of this structure by high-resolution NMR, the researchers say, should facilitate the rational development of therapeutic agents designed to interfere with SARS-CoV programmed −1 ribosomal frameshifting and should also increase our understanding of how pseudoknots stimulate frameshifting.

